# I Am Not My Thoughts: Introducing Mindfulness-Based CBT in the Daily Routine of a Patient with Digital Addiction

**DOI:** 10.1192/j.eurpsy.2026.11739

**Published:** 2026-07-31

**Authors:** A. Argyriadis, E. Kleanthous, E. Patelarou, A. Patelarou, A. Argyriadi

**Affiliations:** 1 Hellenic Mediterranean University, Evidence based Health, Education and Clinical Protocols Laboratory, Heraklion, Greece; 2 Philips University, Nicosia, Cyprus; 3 Hellenic Meditarranean University; 4 Hellenic Mediterranean University, Heraklion, Greece; 5 Frederick University, Nicosia, Cyprus

## Abstract

**Introduction:**

Digital addiction, particularly in the context of excessive smartphone and social media use, has been associated with impaired cognitive control, emotional dysregulation, and social withdrawal. Cognitive Behavioral Therapy (CBT) is widely recognized for addressing maladaptive thought patterns, while mindfulness-based strategies enhance present-moment awareness and reduce reactivity. This case study explores the integration of mindfulness-based CBT (MB-CBT) in the daily routine of an adult patient presenting with digital dependency and co-occurring symptoms of anxiety and low self-esteem.

**Objectives:**

The objectives of this study are to apply a mindfulness-based cognitive behavioral therapy (MB-CBT) framework in managing digital addiction within psychiatric nursing settings, while examining the extent to which structured mindfulness interventions can enhance self-awareness, strengthen emotional regulation, and contribute to a measurable reduction in screen time. Furthermore, the study seeks to assess the feasibility of integrating MB-CBT strategies into routine nursing care, providing valuable insights into their potential as sustainable practices in contemporary mental health care.

**Methods:**

A 27-year-old male patient presented with compulsive smartphone use, averaging 8–10 hours/day, interfering with his work performance and relationships. Initial assessment indicated cognitive distortions (e.g., fear of missing out, catastrophizing), sleep disturbances, and social avoidance. A 6-week MB-CBT intervention plan was implemented, focusing on Psychoeducation on cognitive distortions and attentional hijacking. Mindfulness practices (body scan, breath awareness, mindful use of devices), Cognitive restructuring using thought records and Behavioral activation with scheduled offline activities. Data were collected via self-report logs, screen time tracking, and weekly reflections.

**Results:**

By the end of week 6, the patient demonstrated a 45% reduction in daily screen time, improved sleep hygiene, and increased participation in social and physical activities. Thought records revealed decreased fusion with distressing thoughts ("I am missing out," “I’ll be irrelevant”). The patient reported improved ability to observe thoughts non-judgmentally and disengage from automatic behaviors.

**Conclusions:**

Integrating mindfulness-based CBT techniques into nursing care can offer a practical, evidence-based response to emerging behavioral addictions. This case highlights the importance of empowering patients with metacognitive tools to break the cycle of compulsive digital use and improve psychological well-being. Mental health nurses are in a strategic position to deliver MB-CBT within structured care plans, especially in the context of rapidly evolving digital stressors.

**Disclosure of Interest:**

None Declared

